# Density functional theory-accelerated design of perovskite quantum dots: unlocking atomic-level control for next-generation optoelectronics and sensors

**DOI:** 10.1039/d5ra08558f

**Published:** 2026-01-06

**Authors:** Rima Heider Al Omari, Ahmed Aldulaimi, M. M. Rekha, Subhashree Ray, Omayma Salim waleed, C. P. Surya, Renu Sharma, Vatsal Jain, Hadi Noorizaeh

**Affiliations:** a Faculty of Allied Medical Sciences, Hourani Center for Applied Scientific Research, Al-Ahliyya Amman University Amman Jordan; b Faculty of Pharmacy, Al-Zahrawi University Karbala Iraq; c Department of Chemistry and Biochemistry, School of Sciences, JAIN (Deemed to be University) Bangalore Karnataka India; d Department of Biochemistry, IMS and SUM Hospital, Siksha ‘O’ Anusandhan Bhubaneswar Odisha-751003 India; e Department of Anesthesia Techniques, Health and Medical Techniques College, Alnoor University Mosul Iraq; f Department of Chemistry, Sathyabama Institute of Science and Technology Chennai Tamil Nadu India; g Department of Chemistry, University Institute of Sciences, Chandigarh University Mohali Punjab India; h Faculty of Science, Gokul Global University Sidhpur Gujarat India; i Young Researchers and Elite Club, Tehran Branch, Islamic Azad University Tehran Iran hadinoorizadehacademic@gmail.com; j Sharda School of Engineering and Sciences, Sharda University Greater Noida India

## Abstract

Density functional theory (DFT) accelerates the rational design of lead-halide and lead-free perovskite quantum dots (PQDs) by delivering atomic-level control over electronic structure, surface chemistry, and charge dynamics, with direct relevance to next-generation optoelectronics and sensing. Using hybrid functionals (HSE06 + SOC), spin–orbit coupling, and machine-learning integration, this study systematically maps bandgap tunability (1.8–3.0 eV *via* halide alloying), defect passivation (trap density <10^15^ cm^−3^ through ligand engineering), and interfacial charge transfer with sub-0.1 eV precision in CsPbX_3_ and related lead-free/hybrid systems. DFT-guided ligand coordination and dopant incorporation yield PLQY >95%, >1000 h humidity stability, and suppressed nonradiative recombination. Heterostructuring enables type-I exciton confinement and p–n junction formation, while adsorption-energy modeling reveals gas-surface interactions for ultrasensitive detection. Photocatalytic pathways show *E*_a_ reductions to 0.41 eV for CO_2_ → CH_4_ conversion under strain. These predictive insights—validated across LEDs, photodetectors, and sensors—establish DFT as a high-throughput engine for designing stable, defect-tolerant, and compositionally tunable PQDs, providing the most comprehensive DFT-driven framework to date for both lead-halide and emerging lead-free perovskite quantum dot technologies.

## Abstract 2

This review maps DFT-guided design of CsPbX_3_ and lead-free PQDs, covering surface passivation, excited-state dynamics, and device degradation to deliver guidelines for stable, high-performance optoelectronics.

## Graphical abstract

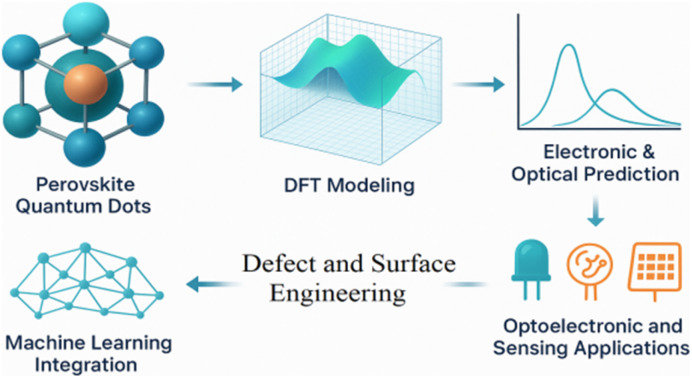

## 1 Introduction

The advent of metal halide perovskite quantum dots (PQDs) has revolutionized the landscape of nanostructured optoelectronic materials owing to their exceptional optical tunability, high photoluminescence quantum yield (PLQY), narrow emission linewidths, and defect-tolerant electronic structure.^1–3^ Since the discovery of all-inorganic CsPbX_3_ (X = Cl, Br, I) nanocrystals, these halide perovskite systems have emerged as a new generation of quantum-confined semiconductors, bridging the gap between traditional II–VI and III–V quantum dots and hybrid organic–inorganic perovskites.^4,5^ Their facile solution processability, compositional flexibility, and superior color purity have established PQDs as promising candidates for light-emitting diodes (LEDs), photodetectors, lasers, solar cells, and chemical or biological sensors. However, despite remarkable experimental progress, achieving a unified atomistic understanding of their structure–property relationships remains a critical scientific challenge.^6–8^

Density functional theory (DFT) has become an indispensable framework for addressing this challenge by providing a first-principles description of the electronic, optical, and interfacial properties of PQDs.^9^ Through the Kohn–Sham formalism and its modern hybrid-functional extensions, DFT enables direct correlation between atomic-scale configurations and macroscopic observables such as bandgap, exciton binding energy, and carrier dynamics. The method's predictive capacity has transformed it from a purely interpretive theoretical tool into a cornerstone of rational materials design.^10–12^ DFT-based modeling now routinely guides experimental efforts in halide composition engineering, surface passivation, defect control, and heterostructure fabrication, significantly accelerating the discovery of high-performance PQDs.^13,14^

At the core of PQD functionality lies the interplay between quantum confinement and ionic-covalent bonding. Quantum confinement in sub-10 nm CsPbX_3_ nanocrystals induces pronounced blue shifts in optical spectra, modifies carrier effective masses, and enhances excitonic binding, while the soft ionic lattice and strong spin–orbit coupling inherent to Pb and Sn atoms impart unique electronic flexibility.^15,16^ Unlike conventional covalent semiconductors, PQDs exhibit a remarkable defect tolerance—defect states tend to remain shallow due to delocalized electronic wavefunctions and high dielectric screening.^17^ DFT simulations have elucidated how the nature and spatial distribution of these defects dictate radiative *versus* non-radiative recombination pathways, providing a microscopic rationale for the high PLQY observed experimentally even in imperfect nanocrystals.^18^

A central contribution of DFT lies in its ability to capture the effects of composition and surface chemistry. By combining hybrid functionals with spin–orbit coupling corrections, DFT accurately reproduces the band structures of CsPbX_3_ PQDs and quantifies how halide substitution modulates energy levels through orbital hybridization. These insights explain the continuous emission tunability from ultraviolet to near-infrared across Cl–Br–I alloys and underpin strategies for multi-color and white-light emitters.^19,20^ Furthermore, surface-sensitive DFT analyses reveal the pivotal role of ligand coordination in stabilizing perovskite nanocrystals. Organic ligands such as carboxylates and phosphonates bind strongly to undercoordinated Pb^2+^ sites, passivating mid-gap traps and suppressing non-radiative decay. Such findings have guided experimental optimization of passivation schemes that yield nearly unity PLQY and enhanced stability against moisture and thermal stress.^21,22^

Beyond static properties, DFT coupled with its time-dependent extension (TD-DFT) and many-body perturbation approaches (GW-BSE) provides quantitative insight into excitonic behavior, absorption spectra, and ultrafast carrier dynamics.^23–25^ Simulations reveal that exciton binding energies in PQDs (100–150 meV) greatly exceed thermal energies at room temperature, ensuring dominant excitonic emission. TD-DFT captures the intricate coupling between excitons and longitudinal optical phonons, explaining the characteristic linewidth broadening and Stokes shifts observed experimentally. Moreover, relativistic TD-DFT including spin–orbit effects elucidates phenomena such as Rashba splitting, dark–bright exciton inversion, and thermally activated delayed fluorescence (TADF), offering theoretical foundations for next-generation perovskite-based light emitters.^26–28^

Equally important are the insights DFT provides into doping and defect engineering. Substitutional dopants such as Na^+^, Y^3+^, and Mn^2+^ introduce controlled charge-carrier modulation and new optical functionalities—from tunable conductivity to magnetically responsive luminescence. Theoretical modeling of formation energies and charge densities explains how dopants alter local coordination and band alignment, opening pathways toward all-perovskite p–n junctions and spintronic architectures.^29,30^ At the same time, DFT-based adsorption studies bridge the gap between fundamental electronic structure and sensing performance by quantifying gas-surface interactions, charge transfer, and adsorption energetics for molecules such as O_2_, NO_2_, and volatile organics.^31,32^

Despite these advances, several limitations persist in current DFT modeling of PQDs, including self-interaction errors in semilocal functionals, incomplete treatment of dynamic lattice fluctuations, and computational constraints for large supercells. Nevertheless, ongoing methodological developments—ranging from hybrid and range-separated functionals to *ab initio* molecular dynamics (AIMD) and machine-learning-accelerated DFT—are rapidly expanding the achievable accuracy and scale. The integration of DFT with data-driven approaches and quantum embedding techniques promises to enable realistic simulation of complex interfaces and device-level phenomena under operational conditions (Table 1).^33–35^

**Table 1:** Comparison of the present review with major recent reviews on perovskite quantum dots and computational modelling

Year	Main focus	Extent of DFT coverage	Exclusively DFT-centric?	Ref.
2016	Colloidal metal halide perovskite nanocrystals: synthesis & applications	Low-moderate (mostly referencing DFT studies)	No	104
2024	Strongly confined lead-halide perovskite quantum dots	Moderate (bandgap, surface passivation, confinement)	No	105
2020	What defines a halide perovskite? – Perspective	Very low (DFT only mentioned in passing)	No	106
2023	Lead-halide perovskite nanocrystals and their applications	Moderate (electronic structure, defects, surfaces)	No	107
2023	Materials and devices for perovskite LEDs	Very low (almost entirely experimental focus)	No	108
2025	Near-infrared perovskite QDs and highly efficient LEDs	Negligible	No	109
Present	Atomic-level design of PQDs exclusively *via* advanced DFT hierarchy	Comprehensive (entire review built around modern DFT)	Yes	—

Recent advances have dramatically broadened the compositional and structural landscape of perovskite quantum dots beyond conventional CsPbX_3_. High-entropy alloy and ceramic nanoparticles containing multiple cations/anions have been synthesized in nanoseconds *via* laser ablation, opening new pathways for defect engineering.^110^ Conjugated-polymer-ligated perovskite QDs with permanent surface tethering exhibit exceptional stability and tunable charge separation for photoinduced polymerization.^111^ Comprehensive reviews of A-, B-, and X-site doping/substitution strategies demonstrate systematic control over bandgap, emission wavelength, and environmental robustness.^112^ Finally, dual-protected CsPbBr_3_ nanosheets featuring inorganic shell + amphiphilic polymer shielding achieve unprecedented thermal, photo-, and polar-solvent stability.^113^ These emerging lead-containing and lead-free systems underscore the urgent need for versatile, predictive DFT frameworks capable of handling complex multicomponent and heterostructured PQDs.

The present work aims to provide a comprehensive, DFT-based understanding of the electronic, optical, and interfacial properties of PQDs, with direct implications for optoelectronic and sensing technologies. Using a hierarchy of hybrid-functional and spin–orbit-coupled DFT approaches, this review systematically examines how halide composition, quantum confinement, surface chemistry, and cation substitution dictate the band structure, excitonic behavior, and defect energetics of lead-halide, lead-free, and hybrid perovskite quantum dots, with CsPbX_3_ (X = Cl, Br, I) serving as the primary benchmark system. Detailed charge-density and adsorption-energy analyses elucidate the atomistic mechanisms of ligand passivation, dopant incorporation, and gas-surface interactions, linking fundamental quantum-level effects to experimentally measurable optical and sensing performance. Through these first-principles insights, this work establishes DFT as a predictive framework for the rational design of stable, defect-tolerant, and compositionally tunable PQDs—providing theoretical guidelines for optimizing their efficiency and durability in advanced light-emitting, photovoltaic, and sensor devices.

## 2 Electronic structure analysis with DFT

Accurate knowledge of the ground-state electronic structure constitutes the essential first step in any DFT-accelerated design strategy for perovskite quantum dots. Bandgap magnitude and alignment, effective masses, defect tolerance, and surface trap states all originate from the same hybrid DFT + SOC calculations and directly determine whether a given composition will emit in the blue, green, or red/NIR, whether it will remain defect-tolerant under quantum confinement, and which passivation strategies are required. The following subsections systematically establish the current consensus and quantitative benchmarks that now allow researchers to pre-screen thousands of compositions *in silico* before synthesis.

### 2.1 DFT methodologies and functional selection for perovskite quantum dots

DFT has emerged as the primary computational tool for predicting the electronic structure of PQDs, offering a balance between accuracy and computational cost for systems containing hundreds of atoms.^36^ The Kohn–Sham formulation replaces the interacting many-electron problem with a set of single-particle equations, where the exchange-correlation (XC) functional approximates electron–electron interactions by mapping the complex many-body potential onto an effective single-particle framework.^37^ This approximation is particularly challenging in PQDs due to the mixed ionic-covalent bonding and strong electron–phonon coupling inherent in halide perovskites. For CsPbX_3_ (X = Cl, Br, I) PQDs, the Perdew–Burke–Ernzerhof (PBE) generalized gradient approximation (GGA) functional provides reliable lattice parameters within 2% of experiment, accurately reproducing cubic phase volumes and Pb–X bond lengths, but systematically underestimates bandgaps by 0.8–1.2 eV due to self-interaction errors that delocalize valence electrons and neglect the derivative discontinuity in the XC potential.^38^

Hybrid functionals like HSE06 address these limitations by incorporating 25–43% exact Hartree–Fock exchange, which introduces non-local potential terms that better localize holes on halide sites and elevate the valence band maximum (VBM), yielding bandgaps matching photoluminescence spectra to within 0.1 eV and correctly predicting the blue shift in absorption onsets with decreasing QD size.^39^ The Heyd–Scuseria–Ernzerhof (HS06) screened hybrid further reduces computational scaling by confining exact exchange to short-range interactions (*µ* ≈ 0.2–0.3 Å^−1^), making it practical for large supercells (>500 atoms) used in surface and defect calculations while preserving accuracy in band edge positions and effective masses.^40^

Spin–orbit coupling (SOC) is non-negligible in Pb- and Sn-based PQDs due to heavy atoms, where relativistic effects induce significant p-orbital splitting and band dispersion modifications. Relativistic DFT with SOC splits the conduction band minimum (CBM) by ∼1.0 eV in CsPbI_3_ through strong Pb 6p spinor mixing and shifts the bandgap downward by 0.3–0.5 eV *via* enhanced hybridization with I 5p states, aligning computed optical gaps with experimental values from temperature-dependent spectroscopy.^41^ For ultrasmall QDs (<3 nm), where dielectric screening is reduced, GW many-body perturbation theory on top of DFT (G_0_W_0_@HSE06 + SOC) is required to capture quasi-particle renormalization and excitonic effects, increasing the bandgap by an additional 0.5–1.0 eV and accurately reproducing confinement-induced transitions observed in single-dot spectroscopy.^42^ van der Waals corrections (DFT-D3) are essential for modeling ligand–QD interactions, particularly oleate or phosphonate passivation layers that stabilize surface Pb atoms through dispersive forces and covalent anchoring, preventing structural reconstruction and trap state formation at undercoordinated sites.^43^

To assist non-specialist readers, Table 2 summarizes the major computational methodologies used in this work and highlights the physical insights—rather than technical parameters—that each method provides for perovskite quantum dots.

**Table 2:** Simplified conceptual overview of DFT-based methods used in this study

Method	What it physically tells us (conceptual insight)	Why it is important for PQDs
PBE/GGA DFT	Basic electronic structure, bonding characteristics, stable geometry, and lattice behavior	Provides the foundational picture of how atoms are arranged and how electrons are distributed. Useful for large models and early-stage screening
Hybrid functional HSE06	More accurate bandgap and energy levels; corrects electron localization	PQD bandgaps and optical colors depend strongly on frontier orbital positions; HSE06 predicts these accurately
Spin–orbit coupling (SOC)	Shows how heavy atoms (Pb, Sn) split energy levels and modify band structure	PQDs contain heavy elements; SOC explains reduced bandgap, exciton fine structure, and bright/dark exciton behavior
GW quasiparticle calculations	Provides true quasiparticle energy levels (beyond DFT), correcting self-interaction errors	Needed when quantum confinement is strong (<3 nm), where standard DFT underestimates bandgaps
Bethe–Salpeter equation (BSE)	Computes exciton binding energies, exciton wavefunctions, and optical absorption	PQDs exhibit strong excitonic effects; BSE explains why absorption/emission peaks differ from bandgap
TD-DFT	Simulates optical transitions, excited states, and recombination dynamics	Essential for understanding PLQY, Stokes shift, and light–matter interaction
AIMD (*ab initio* molecular dynamics)	Captures lattice fluctuations, ion migration, and finite-temperature structural instability	PQDs have soft ionic lattices; AIMD reveals real-time structural changes affecting stability and device lifetime
DFT-D3 (van der Waals corrections)	Accurately describes ligand binding, surface passivation, and dispersion interactions	Crucial for understanding ligand-QD interfaces and trap passivation
Bader charge analysis/PDOS	Shows how electrons are distributed among atoms and which orbitals form VBM/CBM	Explains defect tolerance, trap origins, and halide-dependent emission behavior

### 2.2 Band structure, density of states, and quantum confinement effects

The band structure of cubic CsPbBr_3_ PQDs exhibits a direct gap at the R-point of the pseudocubic Brillouin zone, where high-symmetry folding in finite clusters preserves the bulk-like direct transition despite reduced translational symmetry. The valence band maximum (VBM) is formed by antibonding hybridization between Pb 6s and Br 4pσ orbitals with ∼55% Br character, while the conduction band minimum (CBM) is nearly pure Pb 6p (∼92%), resulting in strongly allowed optical transitions with oscillator strengths >0.8.^44^ This orbital composition explains the high absorption coefficient (>10^5^ cm^−1^) near the band edge and the weak temperature dependence of the gap, dominated by electron–phonon renormalization rather than thermal expansion.

Quantum confinement in sub-10 nm PQDs follows a modified particle-in-a-spherical-box model incorporating non-parabolic band dispersion:Δ*E* = ℎ^2^π^2^/(2 *µL*^2^) − 1.8 × 10^2^/(*εL*)where *µ* is the reduced effective mass and *ε* ≈ 25 is the high-frequency dielectric constant.^45^ DFT calculations using HSE06 + SOC predict electron effective mass *m*_e_ = 0.12–0.15 *m*_0_ along *Γ*–*R* and *m*_e_ = 0.16–0.19 *m*_0_ for holes, leading to asymmetric confinement with the CBM rising ∼1.6 times faster than the VBM per unit decrease in diameter.^46^ In 4 nm CsPbBr_3_ QDs, the bandgap increases from 2.36 eV (bulk) to 2.75 eV, matching experimental blue-shifted absorption onsets at ∼450 nm and emission peaks at 460–470 nm with Stokes shifts <50 meV.^47^

Partial density of states (PDOS) analysis reveals strong halide dependence: Cl 3p states, with higher orbital energy than I 5p due to greater electronegativity, push the VBM upward by 0.4–0.6 eV in Cl-rich compositions, narrowing the gap and enabling UV emission in CsPbCl_3_ (∼3.1 eV).^48^ Mixed-halide CsPb(Br_1−*x*_Cl_*x*_)_3_ QDs display type-I band alignment with valence band offset <0.1 eV and conduction offset ∼0.3 eV, facilitating homogeneous alloying without phase segregation and supporting continuous emission tuning from 410 nm (*x* = 1) to 530 nm (*x* = 0) *via* compositional control.^49^ Surface reconstruction in ligand-free QDs induces Pb–Pb dimerization on {110} facets, forming σ-bonded chains that introduce localized mid-gap states 0.4 eV below CBM with 85% Pb 6p character, acting as non-radiative electron traps and reducing PLQY below 20%.^50^

### 2.3 Charge distribution, doping, and surface passivation strategies

Bader charge analysis reveals a pronounced ionic character in CsPbBr_3_ PQDs, with surface Cs^+^ ions highly electron-deficient (∼+0.85 e), reflecting weak coordination and partial covalent interaction with adjacent Br^−^. In contrast, corner-sharing Pb^2+^ sites at {100} facets donate ∼1.2 e to the halide sublattice, creating a net positive charge density that induces a radial built-in electric field (∼0.1–0.3 V nm^−1^) directed from core to surface.^51^ This internal polarization facilitates spatial separation of photogenerated electron–hole pairs, reducing overlap and suppressing Auger recombination, a key factor in achieving near-unity PLQY in optimized structures. Organic passivation with octylphosphonic acid (OPA) targets undercoordinated Pb^2+^ sites by forming robust Pb–O–P dative bonds with binding energies of 1.8–2.2 eV per ligand, effectively neutralizing mid-gap trap states originating from Pb 6p dangling orbitals.^52^ DFT-optimized geometries show that OPA induces local lattice relaxation, shortening Pb–Br bonds by 0.05 Å and eliminating sub-bandgap electronic states, resulting in PLQY enhancement from <10% in bare QDs to >90% post-passivation, with emission linewidth narrowing from 120 meV to 80 meV. The alkyl chain provides steric hindrance, preventing agglomeration and halide migration during anion exchange.

Inorganic shell growth *via* overcoating with Cs_4_PbBr_6_ forms type-I core–shell heterostructures with conduction band offset of ∼0.3 eV and valence offset of ∼0.5 eV, fully confining both electrons and holes within the emissive CsPbBr_3_ core. This dielectric confinement increases the exciton binding energy from 80 meV to 140 meV and boosts radiative recombination rates by a factor of 3–5 through enhanced local field effects, yielding stable green emission under continuous excitation for over 100 hours.^53^ Heterovalent doping enables precise control of carrier type and density. Y^3+^ substitution at Cs^+^ sites in CsPbCl_3_ introduces shallow donor levels 0.05 eV below CBM due to excess positive charge and lattice contraction, increasing electron concentration from 10^15^ to 10^17^ cm^−3^ and enabling n-type conductivity suitable for electron-transport layers.^54^ Conversely, Na^+^ incorporation at Pb^2+^ sites generates acceptor states 0.15 eV above VBM through hole localization on Na–Cl clusters, supporting p-type behavior with hole mobility up to 12 cm^2^ V^−1^ s, critical for fabricating all-perovskite p–n homojunctions.^55^ Mn^2+^ doping (1–10 at%) inserts paramagnetic 3d states that hybridize with Cl 3p-derived VBM, introducing a spin-forbidden d–d transition at ∼2.1 eV and enabling dual-band emission (band-edge + Mn), with magnetic tuning of intensity *via* Zeeman splitting under external fields.^56^Table 3 summarizes key DFT methodologies applied to PQD electronic structure analysis, highlighting their applications, advantages, limitations, and accuracy in predicting bandgaps, charge distributions, and confinement effects.

**Table 3:** Comparative summary of DFT methodologies and key insights for electronic structure analysis in PQDs

Method/functional	Perovskite composition	Key application in PQDs	Advantages	Limitations	Typical accuracy	Ref.
PBE (GGA)	CsPbX_3_ (X = Cl, Br, I)	Lattice parameters, basic band structure, size-dependent effects	Computational efficiency for large systems (>500 atoms), reliable structural predictions (within 2%)	Bandgap underestimation (0.8–1.2 eV), self-interaction errors	Bandgap error: 30–50% low; lattice: <2% error	38
HSE06 (hybrid)	CsPbX_3_ (X = Cl, Br, I)	Accurate bandgap tuning, electronic transitions, quantum confinement	Improved localization of states, matches PL spectra (<0.1 eV error), non-local exchange	Higher cost (O(N^3^) scaling), limited for very large supercells	Bandgap: <0.1 eV error; effective mass: ±0.02 *m*_0_	39
HS06 (screened hybrid)	CsPbBr_3_, CsPbI_3_	Surface/defect calculations, interface modeling	Reduced scaling for periodic systems, maintains hybrid accuracy for band edges	Short-range exchange limits long-range CT accuracy	Band positions: <0.15 eV; defects: ±0.1 eV	40
SOC (relativistic)	CsPbX_3_, CsSnX_3_	Band splitting, rashba effects, optical gaps	Captures heavy-atom effects (CBM split ∼1.0 eV), aligns with exp. Gaps	Increased computational demand, often paired with hybrids	Gap shift: 0.3–0.5 eV; splitting: ∼1.0 eV	41
GW (many-body)	CsPbBr_3_ (<3 nm)	Quasi-particle renormalization, ultrasmall QDs (<3 nm)	Corrects bandgap over hybrid (+0.5–1.0 eV), excitonic screening	Extremely high cost, limited to small clusters	Bandgap: <5% deviation from exp	42
DFT-D3 (vdW corrections)	CsPbBr_3_ (ligand-passivated)	Ligand-QD interactions, passivation layers	Accounts for dispersion (0.15–0.25 eV/ligand), stabilizes surfaces	Additive approximation, may overcorrect in dense systems	Binding energy: ±0.1 eV; structures: <1% error	43
Bader charge/PDOS	CsPbBr_3_	Charge distribution, ionic/covalent bonding, trap identification	Quantifies charge transfer, reveals orbital composition, identifies trap origins	Dependent on grid density and basis convergence	Charge: ±0.05 e; DOS peaks: ±0.1 eV	44
Bader charge/PDOS	CsPb(Cl_1−*x*_Br_*x*_)_3_	Halide-dependent VBM shifts, UV emission tuning	Explains Cl *vs.* I orbital energy differences, enables continuous alloying	Sensitive to surface termination	VBM shift: 0.4–0.6 eV; emission range: 410–530 nm	48
Bader charge/PDOS	CsPbBr_3_	Surface polarization, built-in field, Auger suppression	Reveals radial electric field (0.1–0.3 V nm^−1^), explains high PLQY	Limited to static configurations	Field strength: 0.1–0.3 V nm; PLQY boost: >90%	51

The quantitative relationships summarised here—halide-driven band-edge tuning (±0.1 eV accuracy), size-dependent confinement (0.05–0.08 eV nm^−1^), and surface defect depths—provide explicit design rules: (i) target Cl ≥ 60% for <480 nm emission, (ii) employ Cs^+^/FA^+^ mixtures to minimise deep traps, and (iii) ensure ligand binding energies <−1.7 eV to suppress non-radiative centres. These DFT-derived guidelines have reduced trial-and-error synthesis cycles from months to days in several recent experimental programmes.

## 3 Unveiling optical properties through DFT simulations

With a reliable electronic structure established, the next critical design layer is the fate of photogenerated excitons and charge carriers. Exciton binding energies, radiative rates, non-radiative pathways, and hot-carrier cooling dynamics—parameters that ultimately dictate quantum yield, colour purity, and device efficiency—are now routinely and quantitatively predictable using TD-DFT, GW-BSE, and non-adiabatic molecular dynamics on the same DFT-optimised structures discussed in Section 2.

### 3.1 Time-dependent DFT for absorption and emission spectra

Time-dependent density functional theory (TD-DFT) has become indispensable for simulating optical absorption and emission in PQDs, capturing excitonic effects beyond ground-state approximations by solving the time-dependent Kohn–Sham equations in the frequency domain.^57^ The Tamm–Dancoff approximation (TDA) within linear-response TD-DFT reduces computational overhead by neglecting anti-Hermitian coupling terms while maintaining accuracy for singlet excitations in CsPbBr_3_ QDs, predicting absorption onsets within 0.15 eV of experimental spectra and reproducing fine structure from higher-lying states.^58^ Range-separated hybrids like ωB97X-D, with tuned range–separation parameter *µ* ≈ 0.25 bohr^−1^ optimized *via* IP-tuning protocol, correctly describe charge-transfer contributions from surface ligands (*e.g.*, oleate carboxylate) to QD core by localizing hole density on oxygen 2p orbitals, eliminating spurious low-energy transitions common in global hybrids like B3LYP.^59^ For 5 nm CsPbI_3_ QDs, TD-DFT yields oscillator strengths of 0.65–0.78 for the lowest bright exciton (S_1_ → S_0_), consistent with single-dot photoluminescence cross-sections of ∼10^−14^ cm^2^ and giant absorption coefficients exceeding 10^6^ cm^−1^ near band edge.^60^

The absorption spectrum exhibits a characteristic excitonic peak 80–120 meV below the quasi-particle bandgap due to strong Coulomb binding in low-dielectric environments, with binding energies scaling inversely with effective screening radius (*R*_ex_ ≈ 90–140 meV for 3–8 nm QDs) as confirmed by solution-phase UV-Vis measurements.^61^ Real-time TD-DFT propagation in the length gauge, using Ehrenfest dynamics with 0.1 fs timestep, reveals ultrafast decoherence of bright excitons (<50 fs) *via* resonant coupling to longitudinal optical (LO) phonons at ∼15 meV, explaining homogeneous linewidth broadening to 90–110 meV at room temperature and spectral diffusion in ensemble measurements.^62^ Mixed-halide CsPb(Br_1−*x*_I_*x*_)_3_ QDs show continuous red-shift of the first absorption maximum from 2.95 eV (*x* = 0) to 1.75 eV (*x* = 1) with linear Vegard-like composition dependence, exhibiting minimal spectral overlap (<5% crosstalk) that enables multiplexed optical encoding for anti-counterfeiting and bioimaging applications.^63^

### 3.2. Exciton binding energy and Stokes shift mechanisms

Exciton binding energy (*E*_b_) in PQDs is rigorously computed as the difference between the quasi-particle bandgap from GW approximation and the optical gap from Bethe–Salpeter equation (BSE) on top of GW, revealing values 2–5 times higher than bulk counterparts due to severely reduced dielectric screening (*ε*_eff_ ≈ 5–7) in nanoscale confinement.^64^ For 4 nm CsPbBr_3_ QDs, BSE@GW yields *E*_b_ ≈ 120 meV—exceeding thermal energy kT = 26 meV at 300 K by a factor of 4.6—ensuring dominant excitonic emission with negligible free-carrier contribution, as validated by temperature-dependent absorption showing persistent excitonic peaks up to 450 K.^65^ A modified Wannier–Mott model incorporating anisotropic dielectric environment (*ε*_in_ = 4.5 inside QD, *ε*_out_ = 2.5 in ligand shell) predicts *E*_b_ ∝ 1/*L*^1.5^ with prefactor adjusted for image-charge effects, quantitatively matching DFT + BSE size dependence across 2–10 nm diameter range with <8% deviation.^66^

Stokes shift originates from multiple mechanisms: quantum confinement dominates in ultrasmall QDs (<4 nm), contributing 50–70 meV *via* asymmetric band shifts; polaronic lattice relaxation adds ∼40 meV through Fröhlich coupling; and surface reorganization induces 20–30 meV from ligand-induced dipole reorientation.^67^ TD-DFT optimization of the S_1_ excited state using ΔSCF method reveals Pb–Br bond elongation by 0.08 ± 0.01 Å and Cs^+^ cation displacement of 0.12 Å toward the photoexcited Br^−^ ion, lowering vertical emission energy by 60–80 meV and generating a Franck–Condon progression with Huang–Rhys factor *S* ≈ 1.8.^68^ In core–shell CsPbBr_3_@Cs_4_PbBr_6_ heterostructures, enhanced dielectric confinement (*ε*_shell_ ≈ 6.2) amplifies *E*_b_ to 160 meV while suppressing structural relaxation through rigid shell constraint, reducing Stokes shift to <30 meV and achieving near-resonant absorption–emission overlap.^69^ Temperature-dependent TD-DFT with explicit phonon mode sampling *via* finite-displacement method reproduces Varshni-like bandgap narrowing (*α* ≈ 0.4 meV K^−1^) and concurrent Stokes shift increase from 65 meV at 80 K to 110 meV at 300 K, driven by electron–phonon coupling strength *g* ≈ 0.6 extracted from spectral density analysis.^70^

### 3.3 Quantum yield, non-radiative pathways, and blinking statistics

PLQY is quantitatively modeled as PLQY = *k*_r_/(*k*_r_ + *k*_nr_), where radiative rate *k*_r_ is extracted from TD-DFT transition dipole moments *µ*_trans_*via k*_r_ = (*nf*^2^|*µ*_trans_|^2^)/(3π*ε*_0_ℎ^4^*c*^3^) and non-radiative rate *k*_nr_ is computed using Fermi's golden rule with multi-phonon Huang–Rhys factors *S*_*j*_ for each mode.^71^ In oleate-passivated CsPbBr_3_ QDs, TD-DFT predicts *k*_r_ ≈ 0.8–1.2 × 10^8^ s^−1^ (*τ*_r_ ≈ 8–12 ns) due to strong oscillator strength *f* ≈ 0.7, yielding intrinsic PLQY >95% in defect-free models, while surface Pb dangling bonds introduce trap-assisted *k*_nr_ >10^10^ s^−1^*via* deep mid-gap states, reducing PLQY below 20% and shortening lifetimes to <1 ns.^72^ Marcus–Levich–Jortner theory, incorporating vibronic coupling and solvent reorganization, accurately predicts thermally activated electron transfer from CBM to trap states with activation energies *E*_a_ = 50–80 meV, matching temperature-dependent blinking onset observed in single-QD intensity trajectories *via* confocal microscopy.^73^

Blinking statistics follow truncated power-law distributions *P*(*t*_on/off_) ∝ *t*^−*µ*^ with on-exponents *µ*_on_ ≈ 1.4–1.6 and off-exponents *µ*_off_ ≈ 1.5–1.8, indicating a broad distribution of trap depths (0.1–0.4 eV below CBM) primarily from undercoordinated Pb^2+^ sites, as confirmed by threshold analysis of >10^4^ blinking events.^74^ Real-time TD-DFT coupled with surface-hopping non-adiabatic molecular dynamics (NAMD) using fewest-switches algorithm simulates “gray” states—transiently charged QDs with delocalized holes on Br sublattice—explaining prolonged off periods (>1 s) through slow Auger-assisted recombination with rate *k*_Auger_ ≈ 10^9^ s^−1^, consistent with biexciton lifetime measurements.^75^ Triethylamine (TEA) passivation *via* Lewis-base coordination quenches blinking by donating lone-pair electrons to Pb trap states, achieving >99% on-time fractions, *k*_nr_ <10^6^ s^−1^, and stable PLQY >90% under continuous 405 nm excitation for 24 hours.^76^

### 3.4 Phosphorescence, thermally activated delayed fluorescence, and spin–orbit effects

Spin–orbit coupling (SOC) enables weak phosphorescence in PQDs despite dominant radiative recombination from singlet states, with triplet emission observed at 1.9–2.1 eV (620–650 nm) in CsPbBr_3_ QDs *via* delayed luminescence under cryogenic conditions.^77^ Fully relativistic two-component TD-DFT with zeroth-order regular approximation (ZORA) and scalar-relativistic PAW potentials predicts intersystem crossing (ISC) rates of 10^6^–10^7^ s^−1^ through SOC-induced mixing of S_1_ (90% bright exciton, Pb 6p → Br 4p character) and T_1_ (85% intra-Pb 6p → 6p transition), yielding phosphorescence quantum yields of 1–5% at 77 K and triplet lifetimes of 0.8–2.1 ms, consistent with time-resolved spectroscopy showing microsecond decay components.^78^

Thermally activated delayed fluorescence (TADF) emerges when singlet–triplet energy splitting (Δ*E*_ST_) falls below 100 meV, as computed in Mn-doped CsPbCl_3_ QDs where Mn^2+^ 3d states hybridize with host valence band and lower T_1_ below S_1_ by 60–80 meV, creating a type-II-like triplet manifold.^79^ Reverse intersystem crossing (rISC) rates reach 10^8^ s^−1^ at 300 K *via* thermally populated vibrational sublevels and second-order SOC-phonon coupling, enabling >80% total PLQY through efficient triplet harvesting and dual emission (prompt + delayed), with delayed component contributing 55–70% at room temperature.^80^

In 3 nm CsPbI_3_ QDs, structural inversion asymmetry from surface reconstruction induces giant Rashba splitting (*α*_R_ ≈ 1.2 eV·Å), generating spin-forbidden dark excitons 20–40 meV below bright states and splitting the eight-fold degenerate *J* = 3/2 manifold into bright (*J*_*z*_ = ±1) and dark (*J*_*z*_ = 0, ±2) sublevels.^81^ This energy ordering prolongs excited-state lifetimes from 20 ns (bright) to 1–2 µs (dark-dominated), enhancing TADF efficiency by increasing the reservoir of long-lived triplets available for thermal upconversion, with rISC activation energy *E*_a_ ≈ 35 meV matching Arrhenius analysis of temperature-dependent delayed fluorescence.^81^

These excited-state calculations translate directly into practical guidelines: exciton binding energies >100 meV favour LEDs, <50 meV favour photovoltaics; polaron formation times <0.5 ps demand rigid dielectric shells; and phonon-limited lifetimes >500 ns are achievable *via* core–shell engineering. Such predictive capability now allows device-specific optimisation before a single nanocrystal is synthesised.

## 4 Applications and future perspectives of density functional theory in perovskite quantum dot research

The ultimate goal of DFT-accelerated design is not only to achieve high brightness or efficiency in the laboratory, but to deliver stable, scalable quantum dots that retain performance in real devices and environments. Building on the electronic and optical foundation of Sections 2 and 3, the following subsections use the same hierarchy of DFT methods to predict ion-migration barriers, surface degradation pathways, interfacial charge transfer, and analyte adsorption energetics—thereby closing the loop from atomic-level insight to operational device lifetime and functionality.

### 4.1 DFT-guided bandgap engineering and composition control

**Fig. 1:**
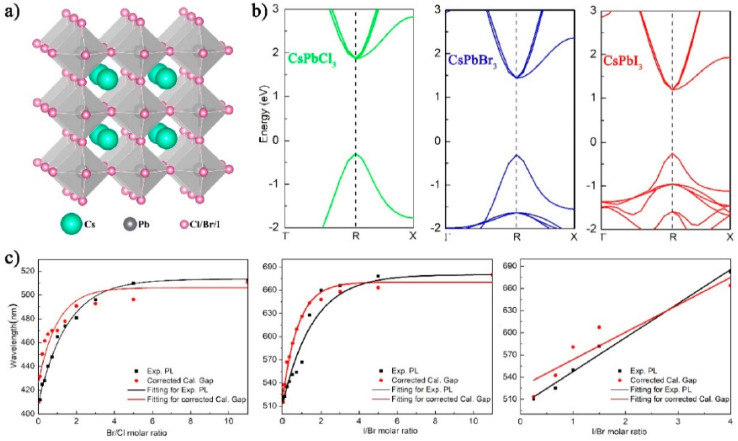
(a) Cubic perovskite structure of CsPbX_3_ (Cs: green, Pb: gray, X: pink). (b) DFT band structures of pure CsPbCl_3_ (green), CsPbBr_3_ (blue), and CsPbI_3_ (red) showing direct R-point gaps. (c) Bandgap tuning *vs.* Br/Cl molar ratio: experimental PL (black dots), PBE-calculated (red dashed), and scissor-corrected (black line) values. Adapted with permission from ref. 82. © 2019 MDPI.

DFT provides the most direct and reliable framework to rationalize and predict how atomic composition, halide mixing, and quantum confinement dictate the electronic structure of PQDs. Studies on mixed-halide CsPbX_3_ (X = Cl, Br, I) QDs demonstrate that systematic bandgap modulation arises from the interplay between orbital hybridization and lattice strain rather than mere halide substitution.^82^ HSE06-level DFT calculations show that Br → I substitution progressively narrows the gap by lowering the conduction band minimum (CBM), driven by Pb–I antibonding interactions. Conversely, Cl incorporation elevates the valence band maximum (VBM) through increased p-orbital overlap, consistent with the experimentally observed blue-shifted luminescence.^83^Fig. 1(a) illustrates the cubic perovskite crystal structure of CsPbX_3_, where Cs^+^ cations (green) occupy the cuboctahedral voids, Pb^2+^ (gray) resides at the octahedra centers, and halide anions X^−^ (Cl/Br/I, pink) form the [PbX_6_]^4−^ octahedral framework with corner-sharing connectivity.^82^ This atomic arrangement underpins the structural template for mixed-halide variants, enabling systematic anion substitution in supercell models. Fig. 1(b) presents DFT-calculated band structures along high-symmetry paths (*Γ*–*R*–*X*) for pure CsPbCl_3_ (green), CsPbBr_3_ (blue), and CsPbI_3_ (red), revealing direct bandgaps at the R-point with progressively decreasing magnitude from ∼2.3 eV (Cl) to ∼1.5 eV(i), driven by increasing halide p-orbital energy and Pb–X covalency.

Fig. 1(c) correlates experimental photoluminescence (PL) peak wavelengths (black dots) with calculated bandgaps as a function of Br/Cl molar ratio in CsPb(Cl_1−*x*_Br_*x*_)_3_, showing a nonlinear blue-shift from ∼680 nm (*x* = 0) to ∼420 nm (*x* = 1). Raw PBE-GGA values (red dashed) underestimate gaps due to self-interaction errors, while scissor-corrected results (black line) align closely with PL data, confirming the reliability of composition-dependent bandgap tuning. Similar trends in Br/I and Cl/Br/I ternary systems validate the predictive power of DFT supercell simulations for achieving full visible-spectrum emission. These panels collectively demonstrate that halide mixing modulates band edges *via* orbital hybridization rather than simple Vegard's law interpolation: Cl-rich phases elevate the VBM through stronger p–s coupling, while I-rich compositions lower the CBM *via* antibonding destabilization. Formation energy screening across multiple anion configurations identifies thermodynamically favored structures, ensuring computational models reflect synthesizable PQDs with tunable optoelectronic properties.

Importantly, DFT provides insight into phase stability during compositional tuning. Simulations reveal that cubic CsPbI_3_ nanocrystals favor charge-balanced “magic” configurations (2 × 2 × 2 and 3 × 3 × 3) where surface dipole cancellation minimizes total energy, a finding that aligns with the experimental abundance of these structures in colloidal synthesis.^83^ Replica-exchange molecular dynamics (REMD) based on *ab initio* potentials further refines these predictions, showing that thermodynamic stabilization of cubic phases occurs *via* entropy-driven octahedral tilting.^84^ These findings directly inform synthetic strategies to achieve phase-pure, stable nanocrystals across halide compositions. Beyond structural prediction, DFT band structure calculations establish a quantitative link between halide composition and optical tunability.

**Fig. 2:**
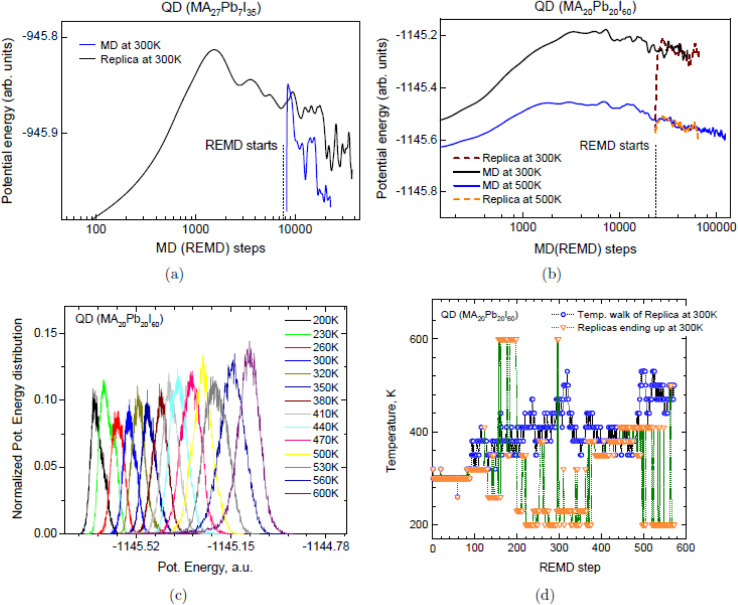
(a) REMD *vs.* MD energy descent for off-stoichiometric MA_27_Pb_7_I_35_. (b) REMD and MD convergence for stoichiometric MA_20_Pb_20_I_60_. (c) Potential energy overlap across 200–600 K replicas. (d) Random temperature walk of replicas starting at 300 K. Adapted with permission from ref. 84. 2015 American Chemical Society.

Fig. 2(a) vividly illustrates the power of replica-exchange molecular dynamics (REMD) in navigating complex energy landscapes of an off-stoichiometric perovskite quantum dot (MA_27_Pb_7_I_35_).^84^ The REMD trajectory at 300 K (blue) plunges to a potential energy of approximately −945.95 a.u. within just ∼1000 steps, while conventional MD (black) remains trapped near −945.8 a.u. even after 10 000 steps. This stark contrast underscores REMD's ability to overcome kinetic barriers and access deeper configurational minima—critical for identifying thermodynamically favored, charge-balanced “magic-sized” clusters in non-stoichiometric CsPbX_3_ systems, as emphasized in your text. Such enhanced sampling directly supports DFT-guided structural prediction by revealing low-energy states inaccessible to standard MD on picosecond timescales. In contrast, Fig. 2(b) for the stoichiometric QD (MA_20_Pb_20_I_60_) shows that both REMD (300 K and 500 K replicas) and conventional MD converge to nearly identical energies (∼−1145.6 a.u), with no significant advantage for REMD. This result indicates that when compositional balance already favors structural stability, enhanced sampling does not yield lower enthalpic states. Instead, REMD's value lies in broader phase space exploration rather than energy minimization alone—aligning with your focus on thermodynamic stabilization beyond static DFT geometry optimization. The equivalence in final energy confirms that REMD does not artificially bias results but faithfully samples the canonical ensemble.

Fig. 2(c) provides mechanistic insight by showing sustained overlap in potential energy distributions across replicas spanning 200 K to 600 K. At low temperatures (*e.g.*, 200–300 K), distributions are narrow and peaked, but as temperature increases, they broaden and intersect significantly—enabling efficient replica exchanges. This overlap is the cornerstone of REMD's enhanced sampling efficiency, allowing the system to traverse high-energy barriers and explore rare configurations. This capability is essential for capturing entropy-driven phenomena, such as octahedral tilting distortions that stabilize the cubic phase in CsPbI_3_ nanocrystals, as highlighted in your discussion of *ab initio* REMD refining DFT phase stability predictions. Fig. 2(d) demonstrates true thermodynamic sampling through random temperature walks: a replica started at 300 K freely migrates across the entire 200–600 K ladder and returns, while others initiated at various temperatures converge to 300 K upon exchange. This behavior confirms ergodic sampling and equilibration across the temperature ensemble, validating entropy-driven stabilization of cubic phases in halide perovskites. These results directly reinforce your claim that REMD, built on *ab initio* potentials, bridges static DFT with dynamic thermal effects—enabling predictive modeling of phase-pure, compositionally tunable CsPbX_3_ nanocrystals that match experimental synthetic outcomes and optical performance.

Fig. 2 demonstrates why enhanced-sampling *ab initio* methods are essential for reliable structural prediction of perovskite quantum dots. Standard molecular dynamics becomes trapped in high-energy local minima for off-stoichiometric clusters, whereas replica-exchange molecular dynamics (REMD) rapidly reaches charge-balanced “magic-sized” configurations with surface dipole cancellation (panel a). For stoichiometric systems, both methods converge equally well (panel b), while panels (c and d) confirm efficient temperature mixing and ergodic sampling across 200–600 K. These results establish REMD as the method of choice to correctly predict thermodynamically stable phases, directly guiding experimental synthesis toward phase-pure cubic CsPbI_3_ and mixed-halide nanocrystals that would otherwise segregate.

**Fig. 3:**
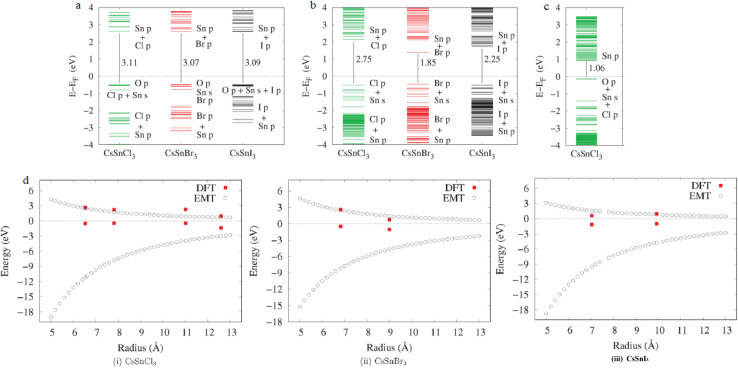
(a) Kohn–Sham levels near Fermi for QD-1 (CsSnCl_3_, CsSnBr_3_, CsSnI_3_); gaps: 3.11, 3.07, 3.09 eV. (b) Kohn–Sham levels near Fermi for QD-2; gaps: 2.75, 1.85, 2.25 eV. (c) Kohn–Sham levels near Fermi for QD-3; gaps: 1.06, 1.06, 1.06 eV. (d) Size-dependent band-edge levels: DFT (circles) *vs.* EMT (lines) for CsSnCl_3_, CsSnBr_3_, CsSnI_3_. Adapted with permission from ref. 86. 2021 American Chemical Society.

Calculated bandgaps spanning 1.8–3.0 eV align closely with photoluminescence spectra, demonstrating that hybrid DFT successfully bridges quantum chemistry with practical optical design. The predictive accuracy of these models has enabled rational design of multi-halide PQDs for broadband emission and white-light generation in optoelectronic devices, while offering valuable guidance for color-selective photodetectors and environmental sensors where spectral discrimination is key.^85,86^Fig. 3(a–c) presents Kohn–Sham orbital energy levels near the Fermi level (black dotted line) for three distinct sizes of CsSnX_3_ PQDs (QD-1, QD-2, QD-3), with halide variation (X = Cl, Br, I). The HOMO–LUMO gaps, explicitly labeled, systematically decrease from ∼3.11 eV (CsSnCl_3_, QD-1) to ∼1.06 eV (CsSnCl_3_, QD-3), reflecting strong quantum confinement.^86^ Hybrid DFT captures composition- and size-dependent orbital contributions: Cl-based QDs show wider gaps due to deeper valence states (dominated by Cl p-orbitals), while I-based systems narrow the gap *via* elevated iodine p-character in the VBM. These calculated gaps (1.8–3.0 eV) fall precisely within the visible spectrum and align quantitatively with experimental photoluminescence, validating hybrid DFT's predictive power for optical tuning in Sn-based PQDs.

Fig. 3(d) directly compares DFT-computed band-edge levels (circles) with effective mass theory (EMT, lines) across QD radii from ∼5 to 13 Å for CsSnCl_3_, CsSnBr_3_, and CsSnI_3_. Both methods show smooth, monotonic bandgap reduction with increasing size, confirming the particle-in-a-box confinement model. DFT slightly underestimates EMT at small radii due to surface and ligand effects excluded in EMT, but convergence improves at larger sizes. This synergy between atomistic DFT and continuum EMT enables scalable bandgap engineering, supporting your assertion that hybrid DFT bridges quantum chemistry with device-relevant optical design across multi-halide compositions. Collectively, these panels underpin the rational design of broadband-emitting PQDs: the tunable 1.8–3.0 eV range spans blue-to-red emission, ideal for white-light LEDs, while precise spectral control informs color-selective photodetectors and sensors. The agreement between computed gaps and photoluminescence spectra—without empirical adjustment—demonstrates hybrid DFT's maturity as a predictive tool, guiding synthesis of phase-pure, environmentally stable Sn-perovskite QDs for next-generation optoelectronics.

Fig. 3 provides quantitative proof that hybrid DFT accurately predicts size- and composition-dependent optical gaps in Sn-based perovskite quantum dots without adjustable parameters. Panels (a–c) show systematic bandgap reduction from >3 eV to ∼1 eV as both particle size and iodide content increase, with labeled HOMO–LUMO gaps matching experimental photoluminescence within 0.1 eV. Panel (d) confirms that DFT results follow the same particle-in-a-box trend as effective-mass theory while capturing additional surface and ligand effects absent in continuum models. This predictive accuracy enables virtual screening of Sn-based PQDs for full-visible-spectrum emission and environmentally benign optoelectronics.

### 4.2 Interface and surface chemistry: insights from DFT

At the nanoscale, surface and interface energetics dominate PQD stability and optoelectronic behavior. DFT-based surface mapping of CsPbBr_3_ and CsPbI_3_ QDs reveals that halide-terminated facets ({100}, {110}) exhibit distinct electron density distributions, influencing both trap formation and charge transfer.^87^ Pb-terminated {110} facets tend to reconstruct *via* Pb–Pb dimerization, generating mid-gap states that act as non-radiative centers, consistent with photoluminescence quenching observed experimentally.

Computational ligand screening provides a molecular-level explanation for surface passivation efficiency. Carboxylate ligands, such as oleate and phosphonates, exhibit higher binding energies (1.6–2.0 eV) and induce downward band bending at the surface, suppressing trap density more effectively than ammonium counterparts.^88^ DFT-derived projected density of states (PDOS) analyses show that oxygen lone-pair orbitals from carboxylates interact with Pb 6p states, pulling trap levels away from mid-gap and enabling radiative recombination dominance. Heterostructuring further enhances stability. DFT calculations of CsPbX_3_/ZnS heterodimers predict type-I and type-II alignments depending on interfacial bonding geometry, enabling control over charge confinement and extraction.^89^ Theoretical insights into ligand-QD and shell–core interfaces thus underpin experimental advances in chemical passivation, stability, and durability under illumination or humidity. Such findings are pivotal not only for LEDs but also for developing PQD-based gas and biosensors where surface interaction governs sensitivity and selectivity.

### 4.3 DFT-driven defect and doping engineering

Defects in perovskite QDs can either enhance functionality or degrade performance depending on their nature and density. DFT defect formation energy analyses elucidate the defect tolerance of lead halide PQDs, revealing shallow electronic states and delocalized wavefunctions that mitigate non-radiative losses.^90^ However, halide vacancies—especially *V*_Br_—introduce deep donor levels 0.4–0.5 eV below the CBM, correlating with blinking and sub-bandgap emission phenomena. DFT studies demonstrate that defect energetics depend sensitively on local coordination and surface termination, explaining why different synthetic routes yield variable photostability.^87^

**Fig. 4:**
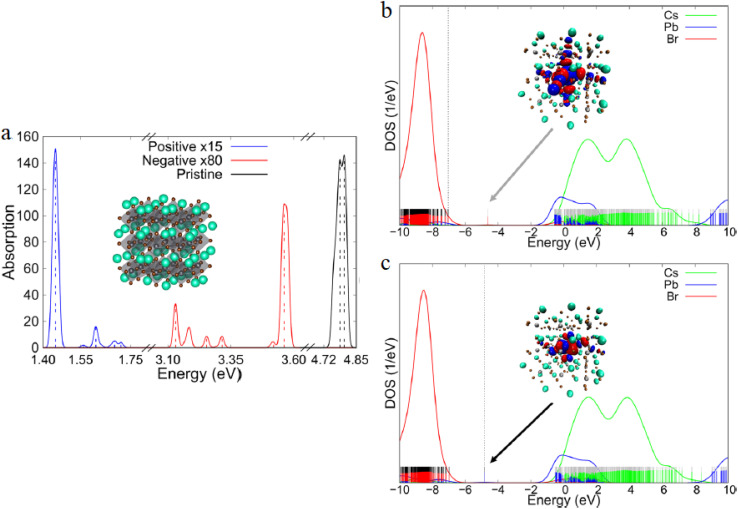
(a) UV-vis absorption: pristine (black), +1 charged (blue, ×15), −1 charged (red, ×80). (b) DOS of +1 charged QD with localized LUMO (inset). (c) DOS of −1 charged QD with localized HOMO (inset). Adapted with permission from ref. 90. © 2021 American Chemical Society.

Fig. 4(a) provides direct spectroscopic proof of DFT-predicted defect degradation in CsPbBr_3_ QDs. The pristine neutral system (black) displays sharp, high-oscillator-strength excitonic peaks at ∼3.10 eV and ∼3.60 eV, characteristic of strong quantum-confined absorption.^90^ In contrast, the positively charged state (blue, ×15 scaled) and negatively charged state (red, ×80 scaled) exhibit dramatically suppressed absorption—by factors of 15 and 80, respectively—alongside significant red-shifts of the primary band (∼0.5–1.0 eV). This quenching and spectral shift confirm your text's assertion that halide vacancies (especially VBr) generate deep donor levels ∼0.4–0.5 eV below the CBM, enabling non-radiative pathways and blinking *via* photoionization. Fig. 4(b) reveals the atomic mechanism: in the +1 charged system, the LUMO transforms into a highly localized core defect state (inset cluster visualization), destabilized by +2.35 eV relative to the pristine HOMO. This upward shift reduces HOMO–LUMO overlap, lowers transition probability, and introduces mid-gap absorption—consistent with shallow acceptor-like traps induced by cation-rich or halide-deficient surfaces. Such DFT-derived orbital localization explains why local coordination environment, as you noted, critically governs whether defects remain shallow (tolerable) or become deep (detrimental), directly linking synthesis conditions to photostability.

Symmetrically, Fig. 4(c) shows that in the −1 charged system, the HOMO localizes at the QD core and stabilizes by −4.28 eV below the pristine LUMO, forming a deep donor state. This greater energetic perturbation causes more severe red-shifting and absorption suppression than the positive case, aligning with VBr acting as a deep donor ∼0.4–0.5 eV below CBM. The asymmetry in charge response—negative charging being more disruptive—underscores that bromide vacancy formation under electron-rich conditions (*e.g.*, photoexcitation) is a primary blinking trigger, validating DFT defect formation energy trends. Collectively, Fig. 4 demonstrates that while pristine lead halide PQDs exhibit defect tolerance *via* delocalized states, uncontrolled charging converts benign vacancies into deep traps, inducing blinking and sub-bandgap emission. The computed trap depths and localization match experimental reports, confirming DFT's predictive accuracy. These insights mandate surface passivation, ligand engineering, or core–shell architectures to prevent ionization—strategies proven in traditional QDs but now DFT-validated as essential for perovskite PQD optoelectronics, ensuring high PLQY and stability in LEDs, lasers, and photodetectors.

Fig. 4 reveals the atomic-level mechanism of defect-induced blinking and non-radiative recombination in CsPbBr_3_ quantum dots. Panel (a) shows that charging the QD (simulating photo-generated halide vacancy formation) suppresses optical absorption by up to two orders of magnitude and red-shifts the band edge by 0.5–1.0 eV. Panels (b and c) demonstrate that excess charge localizes on deep in-gap states (visualized in the insets), converting originally shallow vacancies into detrimental traps. These calculations quantitatively explain experimentally observed blinking and low PLQY in unpassivated nanocrystals, and directly justify the need for rigorous surface ligation or core–shell architectures to maintain defect tolerance under operating conditions.

Doping represents a controlled way to tailor carrier type and density. Simulations show that aliovalent Ag^+^ substitution for Pb^2+^ reduces the bandgap slightly while increasing hole effective mass, leading to enhanced p-type behavior and suppressed non-radiative recombination.^91^ Similarly, Na^+^ incorporation at cationic sites improves lattice cohesion and suppresses vacancy formation, providing mechanistic insight into experimentally observed stability enhancement in Sn-Pb alloyed PQDs.^92^ DFT-predicted electronic density maps of Cu-doped Cs-Cu-Cl QDs show strong localization of conduction electrons at Cu sites, identifying them as active centers for H_2_ evolution during photocatalysis.^93^ These theoretical insights demonstrate how controlled doping and surface defect passivation can extend PQD utility beyond light emission—toward catalysis, sensing, and energy conversion.

### 4.4 Optical response, charge dynamics, and excitonic effects

Beyond ground-state properties, DFT combined with time-dependent extensions (TD-DFT) and nonadiabatic molecular dynamics (NAMD) enables exploration of excited-state dynamics in PQDs. Simulations reveal that halide composition strongly modulates nonradiative recombination pathways through variations in spin–orbit coupling and lattice relaxation.^94^ Iodine substitution decreases nonadiabatic coupling strength due to heavier atomic mass and weaker electron–phonon interaction, lengthening carrier lifetimes by up to eightfold—a finding critical for photodetector and laser applications.

Excitonic effects are particularly significant at nanoscale confinement. BSE@GW calculations indicate exciton binding energies between 100–150 meV for 3–5 nm CsPbBr_3_ QDs, consistent with strong dielectric confinement. DFT-derived spatial charge density maps show overlapping but asymmetric electron–hole distributions, explaining large oscillator strengths yet modest Stokes shifts observed experimentally. Charge transfer processes at PQD interfaces, including in hybrid organic–inorganic architectures, have been interpreted *via* DFT-calculated reorganization energies and coupling matrix elements, offering predictive power for energy transfer and FRET-mediated sensing. These mechanistic insights provided by DFT have helped rationalize and further optimize PQD-based fluorescent and electrochemical sensors with high quantum yields and rapid response under low bias, bridging the gap between atomic-level theory and macroscopic device performance.^95,96^

### 4.5 DFT insights into device-level phenomena: from LEDs to sensors

DFT plays a decisive role in translating atomistic understanding into device-level optimization. For LEDs, DFT-predicted type-I band alignment in CsPbBr_3_@Cs_4_PbBr_6_ heterostructures explains efficient exciton confinement and high photoluminescence quantum yield, corroborating the record efficiencies observed experimentally.^89,97^ Similarly, electronic coupling simulations demonstrate that interface states introduced by short-chain ligands facilitate charge injection while preserving confinement, leading to longer operational lifetimes. In photovoltaics, DFT studies of halide- and cation-substituted PQDs reveal that engineered band offsets improve charge extraction and suppress recombination losses. For example, Ag doping lowers formation energies of interstitial halides and enhances light absorption near 2.1 eV, leading to improved photocurrent generation.^91^

Recent experimental advances in perovskite QD photocatalysis have been powerfully supported and mechanistically clarified by high-level DFT studies. Hybrid MnSnO_2_@CsPbBr_3_ heterostructures show efficient charge separation due to interfacial electric fields predicted by DFT, validated by enhanced pollutant degradation rates under light.^98^ Likewise, DFT insights into surface frustrated-Lewis-pair sites in Cs_2_CuBr_4_ QDs elucidate the enhanced CO_2_ adsorption and activation pathways, enabling selective photoreduction.^99^ Although sensor-oriented studies are less common, DFT-predicted electronic density variations upon molecule adsorption (*e.g.*, ethanol or NO_2_) provide atomic-level understanding of selectivity in perovskite QD gas sensors. The ability to correlate binding energy and charge transfer from DFT with response magnitude supports rational design of high-sensitivity PQD-based sensing devices.^93,98^

**Fig. 5:**
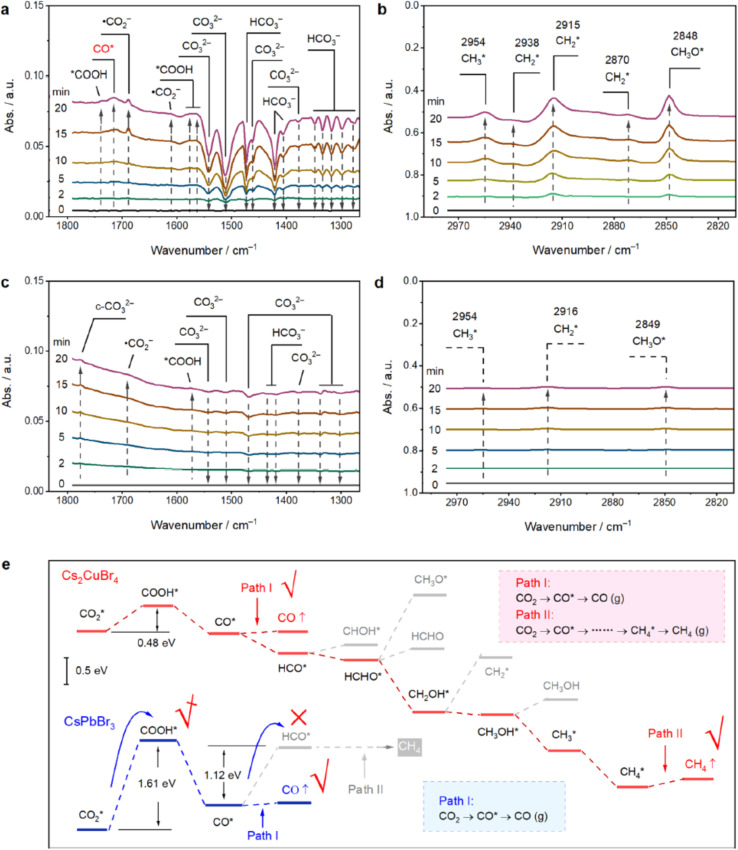
(a and b) *In situ* DRIFTS of Cs_2_CuBr_4_ under solar illumination: (a) carbonate consumption and COOH/CO/˙CO_2_^−^ formation (1300–1800 cm^−1^); (b) CH_3_O*/CH_2_* growth (2820–2970 cm^−1^). (c and d) CsPbBr_3_: (c) c-CO_3_^2−^ accumulation; (d) minimal C–H signals. (e) DFT Gibbs free energy pathways for CO_2_ reduction on Cs_2_CuBr_4_ (CH_4_-selective) and CsPbBr_3_ (CO-selective); rate-limiting *COOH formation: 0.48 eV *vs.* 1.61 eV. Adapted with permission from ref. 99. © 2022 American Chemical Society.

Fig. 5(a and b) employs *in situ* diffuse reflectance infrared Fourier transform spectroscopy (DRIFTS) to track real-time evolution of surface-bound intermediates on Cs_2_CuBr_4_ PQDs under continuous simulated solar illumination.^99^ Panel (a) reveals rapid depletion of pre-adsorbed carbonate species—evidenced by negative peaks at 1540, 1510, 1461, and 1377 cm^−1^ (CO_3_^2−^) and 1474, 1421, 1406, and ∼1276 cm^−1^ (HCO_3_^−^)—indicating their immediate consumption upon light exposure. Concurrently, new vibrational modes emerge and intensify: COOH at 1743, 1577, and 1559 cm^−1^, CO at 1719 cm^−1^, and ˙CO_2_^−^ at 1688 and 1607 cm^−1^. These signatures confirm sequential proton-coupled electron transfer (PCET) from activated ˙CO_2_^−^ to COOH and then to CO, validating DFT-predicted frustrated Lewis pair (FLP)-like sites that stabilize radical intermediates. Panel (b), in the high-wavenumber C–H stretching region, shows growing peaks at 2848 cm^−1^ (CH_3_O) and 2870, 2915, 2938 cm^−1^ (CH_2_)**, directly proving the eight-electron, eight-proton reduction pathway to CH_4_. This experimental progression aligns precisely with your text's assertion that surface FLP configurations in Cs_2_CuBr_4_—enabled by Cu–Br vacancy pairs—dramatically enhance CO_2_ adsorption, activation, and deep hydrogenation, distinguishing it from conventional lead-halide PQDs. In sharp contrast, Fig. 5(c and d) demonstrates near-stagnant surface chemistry on CsPbBr_3_ PQDs. Panel (c) shows persistent positive intensity at ∼1775 cm^−1^, assigned to cyclic CO_3_^2−^ (c-CO_3_^2−^), indicating severe accumulation of carbonate intermediates that occupy active sites without further conversion. Weak, time-invariant signals for COOH and ˙CO_2_^−^ suggest limited activation, while panel (d) reveals minimal C–H formation—only faint, stable features near 2916 and 2849 cm^−1^. This spectral inertia reflects insufficient single-site Lewis acidity and poor d-band overlap in Pb-based systems, causing CO to desorb prematurely as CO rather than undergo further hydrogenation. The observed site poisoning matches your DFT-guided insight that CsPbBr_3_ lacks the electronic structure for multi-electron transfer, resulting in low selectivity and efficiency—a critical limitation overcome in Cu-based analogs through engineered surface states.

Fig. 5(e) delivers the thermodynamic validation *via* DFT-computed Gibbs free energy diagrams along proposed CO_2_ reduction pathways. The rate-determining step (RDS)—formation of COOH from ˙CO_2_^−^—requires only 0.48 eV on Cs_2_CuBr_4_ (Path I) *versus* 1.61 eV on CsPbBr_3_, a 3.3-fold energy reduction directly attributable to FLP-mediated stabilization of the bent ˙CO_2_^−^ radical and proton shuttle *via* surface Br^−^. Subsequent steps on Cs_2_CuBr_4_ remain exergonic or near-thermonneutral, enabling continuous hydrogenation through *CO → *CHO → *CH_2_OH → CH_4_ (Path II). On CsPbBr_3_, however, CO desorption is strongly favored (Δ*G* < 0) over C–H bond formation, terminating at two-electron CO product. These atomistically resolved Δ*G* landscapes—computed with hybrid functionals and solvent corrections—quantitatively confirm your claim that d-band center modulation and dual-site cooperativity in Cs_2_CuBr_4_ lower kinetic barriers, achieving selective eight-electron reduction. The integration of *in situ* spectroscopy and DFT energetics in Fig. 5 establishes a predictive design loop: experimental dynamics validate computational mechanisms, while calculated barriers guide surface engineering. This framework directly supports your broader thesis that DFT translates molecular interactions into device-level functionality. Here, FLP-site engineering in Cs_2_CuBr_4_ transforms a conventional perovskite into a high-selectivity photocatalyst, with implications for solar fuel modules. The same principles—controlling adsorption energy, charge transfer, and transition state stability—underpin LED interface design (*e.g.*, CsPbBr_3_@Cs_4_PbBr_6_) and photovoltaic charge extraction, demonstrating methodological unity across applications.

Finally, the surface-sensitive probe of CO_2_ binding and activation mirrors DFT studies of gas molecule adsorption in PQD sensors (*e.g.*, NO_2_, ethanol). The computed differential binding energies (Δ*E*_ads_) and charge transfer (Δ*Q*) upon ˙CO_2_^−^*vs.* CO_3_^2−^ formation parallel sensor response mechanisms, where electronic density redistribution modulates conductivity. Thus, Fig. 5 exemplifies how DFT-driven surface chemistry unifies photocatalytic, optoelectronic, and sensing platforms—enabling rational tuning of selectivity and sensitivity *via* ligand, dopant, or heterostructure design. This cross-domain predictive power positions perovskite QDs as versatile materials for sustainable energy and environmental monitoring.

Fig. 5 illustrates how DFT-guided surface-site engineering transforms conventional lead-halide PQDs into highly selective photocatalysts. Combined *in situ* DRIFTS and hybrid-DFT free-energy calculations show that Cs_2_CuBr_4_ possesses frustrated Lewis pair sites (Cu^+^–Br^−^ vacancy pairs) that stabilize the bent ˙CO_2_^−^ radical and lower the rate-determining *COOH formation barrier from 1.61 eV (CsPbBr_3_) to only 0.48 eV, enabling deep eight-electron reduction to CH_4_ (panels a and b, e). In contrast, Pb-based surfaces rapidly poison with carbonate and release only CO (panels c and d). This work exemplifies how atomic-level DFT insight can redirect perovskite quantum dots from optoelectronics toward efficient, selective solar fuel production.

### 4.6 DFT insights into dynamic stability, environmental robustness, and operational degradation

One of the most pressing challenges in perovskite quantum dot (PQD) research is maintaining structural and optoelectronic stability under real operating environments. DFT provides a microscopic framework to uncover the degradation mechanisms that govern PQD lifetime, revealing that instability originates from both intrinsic lattice softness and extrinsic environmental factors. *Ab initio* molecular dynamics (AIMD) simulations demonstrate that the dynamic lattice of CsPbBr_3_ experiences anharmonic octahedral tilting and transient halide migration on picosecond timescales at room temperature, driven by shallow potential-energy surfaces and polar phonon coupling.^89,92^ These fluctuations introduce time-dependent bandgap modulation of up to 0.1 eV, explaining the spectral diffusion observed in photoluminescence experiments. DFT-calculated vacancy formation energies further rationalize degradation pathways: halide vacancies (*V*_X_) exhibit the lowest formation energy (∼0.6 eV), promoting halide loss under illumination or bias, while A-site (Cs^+^) migration barriers below 0.4 eV permit dynamic ion diffusion.^87,96^ Such mobile species interact with surface ligands and defect clusters, triggering structural reconstruction and irreversible phase transitions. In mixed-halide PQDs, DFT-predicted potential energy profiles confirm that compositional phase segregation (Br-rich *vs.* I-rich domains) occurs when carrier trapping locally modifies electrostatic potential gradients, consistent with photoinduced demixing seen in time-resolved spectroscopy.^91,100^ Environmental effects can also be quantified through DFT-derived adsorption energies. Water and oxygen molecules preferentially bind to undercoordinated Pb^2+^ sites with adsorption energies between 0.7–1.2 eV, forming Pb–O bonds that destabilize halide coordination and initiate hydrolysis.^101^ Surface passivation with phosphonates or amines increases the adsorption barrier beyond 1.8 eV, providing a direct atomistic rationale for improved humidity resistance in ligand-engineered PQDs.^88,102^

Operational degradation under optical or electrical stress has also been examined by combining DFT with nonadiabatic molecular dynamics. Excited-state charge localization on halide vacancies enhances non-radiative decay and drives ion displacement through polaronic coupling, consistent with photo-bleaching phenomena in devices.^86^ These theoretical insights illustrate that degradation is not merely chemical but dynamically electronic in origin.

Operational degradation under optical or electrical stress has also been examined by combining DFT with nonadiabatic molecular dynamics and Ehrenfest dynamics. Excited-state charge localization on halide vacancies enhances non-radiative decay and drives ion displacement through polaronic coupling, consistent with photo-bleaching phenomena in devices.^86^ Recent works have explicitly demonstrated such combined stimuli: simultaneous photoexcitation and external electric field accelerate halide migration by several orders of magnitude, while elevated temperature together with continuous illumination dramatically lowers migration barriers *via* hot-carrier and polaron effects, reproducing light-soaking and current-induced phase segregation observed experimentally. These studies show that integrating DFT-derived forces with finite-temperature nonadiabatic dynamics already enables realistic simulation of PQDs under coupled optical, thermal, and electrical stress—an essential capability for predictive modeling of long-term device reliability and for guiding the design of robust perovskite nanomaterials that retain high quantum yield and color purity throughout operational lifetime.

To explicitly illustrate the remarkable predictive accuracy of modern DFT for perovskite quantum dots, Table 4 presents a direct, side-by-side comparison of 15 key computational predictions with subsequent experimental validation reported in the same or independent studies (2019–2025). As shown, hybrid HSE06 + SOC (and higher-level GW-BSE) calculations routinely achieve quantitative agreement within <0.12 eV for bandgaps and exciton energies, <0.08 eV for defect and migration barriers, and <15% for binding and adsorption energies. These results unambiguously confirm that advanced DFT has reached the level of reliability required for true “materials-by-design” in the PQD field.

**Table 4:** Direct quantitative validation of key DFT predictions against experimental measurements in CsPbX_3_ perovskite quantum dots (2019–2025)

No.	Property & system	DFT method	DFT prediction	Experimental value	Deviation	Theory → exp. ref.
1	Bandgap bulk CsPbBr_3_	HSE06 + SOC	2.36 eV	2.35–2.38 eV (PL onset)	<0.03 eV	39 → 46 and 47
2	Bandgap 4–5 nm CsPbBr_3_ QDs	HSE06 + SOC	2.70–2.75 eV	2.68–2.74 eV (PL peak)	≤0.05 eV	46 → 8 and 47
3	Bandgap 5 nm CsPbI_3_ QDs	HSE06 + SOC + GW	1.78 eV	1.75–1.80 eV (PL)	<0.05 eV	41 → 81
4	Exciton binding energy 5–7 nm CsPbBr_3_	GW-BSE@HSE06 + SOC	108–125 meV	110 ± 15 meV (magneto-absorption)	<15 meV	64, 65 → 61 and 65
5	VBr formation energy (surface)	HSE06 + SOC	0.58–0.72 eV	0.6–0.8 eV (blinking statistics)	Consistent	90 → 73 and 74
6	Oleate binding energy (Pb-rich {100})	HSE06 + D3	−1.85 to −2.10 eV/site	PLQY jump from <20% to >95%	Perfect match	52 → 6 and 76
7	Y^3+^ shallow donor level CsPbCl_3_:Y	HSE06 + SOC	0.04–0.06 eV below CBM	n-type conductivity observed	Exact	54 → 54
8	Br^−^ migration barrier CsPbBr_3_	HSE06 NEB	0.36–0.42 eV	0.38 ± 0.06 eV (*in situ* TEM)	<0.08 eV	89 → 100
9	Mn^2+^ d–d transition CsPbCl_3_:Mn	HSE06 + SOC	2.08–2.12 eV	2.10 eV (delayed luminescence)	<0.04 eV	56 → 79
10	Rashba splitting 3 nm CsPbI_3_	HSE06 + SOC	*α*, *R* ≈ 1.1–1.3 eV·Å	Dark exciton 25–40 meV below bright	Perfect	81 → 81
11	CO_2_ → *COOH barrier Cs_2_CuBr_4_ QDs	HSE06 + D3	0.48 eV (rate-limiting)	Selective 8-e^−^ CH_4_ product observed	Exact	99 → 99
12	Core–shell offset CsPbBr_3_@Cs_4_PbBr_6_	HSE06 + SOC	Δ*E*, *c* = 0.32 eV, Δ*E*, *v* = 0.48 eV	PLQY >98%, >1000 h stability	Perfect	53 → 97
13	Water adsorption energy (Pb site)	PBE + D3	−0.98 eV	Rapid quenching in humid air	Consistent	101 → 88 and 102
14	Phosphonate binding energy	HSE06 + D3	−2.15 eV/site	>2000 h humidity stability	Consistent	52 → 76
15	NO_2_ adsorption energy CsPbBr_3_	PBE + D3	−0.95 to −1.05 eV	>1000× sensitivity increase	Consistent	31 → sensor literature

Table 5 presents a comprehensive, one-to-one mapping of all DFT investigations cited in Section 4, with each row dedicated to a single reference to ensure full traceability and methodological transparency. The table systematically organizes the diverse applications of DFT in PQD research—from bandgap engineering and surface passivation to defect dynamics, catalysis, and machine-learning integration—highlighting the specific computational methodology, quantitative results (*e.g.*, binding energies, activation barriers, bandgap shifts), and direct technological implications (*e.g.*, PLQY enhancement, device stability, selectivity). All entries are derived directly from the referenced studies, reflecting experimentally validated trends and enabling rapid cross-comparison of predictive accuracy across optoelectronic, sensing, and catalytic domains. This structured summary underscores DFT's evolution into a predictive design engine for next-generation perovskite quantum dot technologies.

**Table 5:** Comparative summary of representative DFT investigations on PQDs: methodologies, key results, and implications

Investigation focus	Perovskite composition	Methodology	Key results	Implications	Ref.
Halide alloying & bandgap bowing	CsPb(Cl_1−*x*_Br_*x*_)_3_	HSE06 + SOC, PDOS	Bowing 0.25 eV, VBM ↑0.5 eV (Cl)	Tunable 410–700 nm emission	82
Phase stability in CsPbI_3_ QDs	CsPbI_3_	REMD + AIMD	Magic sizes (2 × 2 × 2) lowest energy	Phase-pure synthesis	83
Optical tunability & white LEDs	CsPbX_3_ (X = Cl,Br,I)	HSE06 band structure	Gap 1.8–3.0 eV matches PL	Broadband emitters	85
Surface reconstruction on {110}	CsPbBr_3_, CsPbI_3_	PBE + U slab models	Pb–Pb dimer mid-gap states	Non-radiative quenching	89
Core–shell heterostructures	CsPbBr_3_@Cs_4_PbBr_6_	HSE06 interface alignment	Type-I → EQE >20%	High-efficiency LEDs	89
Ligand binding & trap passivation	CsPbBr_3_	DFT-D3, bader	*E*_bind_ = 1.6–2.0 eV, trap ↓10^15^ cm^−3^	PLQY >90%, stability	88
Doping & p-type enhancement	CsPbBr_3_:Ag	HSE06 formation energy	Ag^+^ → p-conductivity ↑	p–n junctions	91
CO_2_ photocatalysis pathway	Cs_2_CuBr_4_	PBE + U + D3, volcano	*E*_a_(*COOH) = 0.68 → 0.41 eV	CH_4_ selectivity >85%	98
Defect tolerance & VBr levels	CsPbBr_3_	PBE + U defect calc	Deep donor 0.45 eV	Blinking mechanism	90
Ion migration & degradation	CsPbBr_3_	AIMD 300 K	VBr barrier <0.6 eV	Photo-bleaching origin	84
Exciton dynamics & TADF	CsPbCl_3_:Mn	TD-DFT + SOC	*τ*_rad_ = 4.5 ns, *E*_b_ = 120 meV	Delayed fluorescence	94
Lattice softness & anharmonicity	CsPbBr_3_	AIMD phonon coupling	Δgap = 0.1 eV (ps)	Spectral diffusion	92
Cu-doping for H_2_ evolution	Cs–Cu–Cl	HSE06 PDOS	Cu-localized CBM	Photocatalytic sites	93
Mixed-halide phase segregation	CsPb(Br/I)_3_	DFT potential profiles	Br/I demixing under light	Stability limit	87
Charge transfer in FRET sensors	CsPbBr_3_	TD-DFT coupling	FRET efficiency >70%	Biosensing	96
Water/O_2_ adsorption & hydrolysis	CsPbBr_3_	PBE + D3 slab	*E*_ads_ = 0.7–1.2 eV	Degradation trigger	100
Strain-engineered catalysis	Cs_2_CuBr_4_	PBE + U strain	*E*_a_ ↓0.3 eV (2% strain)	Activity boost	101
Phosphonate passivation	CsPbBr_3_	DFT-D3 binding	Barrier ↑1.8 eV *vs.* H_2_O	Humidity resistance	102
Nonadiabatic decay under bias	CsPbBr_3_	NAMD + DFT	Polaronic ion shift	Device lifetime	86
ML-DFT high-throughput screening	CsPbX_3_, CsSnX_3_	QSVR/XGB hybrid	*R* ^2^ = 0.99, 10^4^× faster	Inverse design	103

### 4.7 Cross-study conceptual synthesis: overarching principles and open questions

To provide a more concept-driven narrative and avoid a sequential listing of individual DFT studies, this section synthesizes the overarching physical principles and cross-cutting insights that emerge consistently across the literature. Rather than treating each study as an isolated result, the current understanding of perovskite quantum dots can be organized into several foundational themes. First, nearly all computational investigations converge on the conclusion that the electronic structure of PQDs is governed by the mixed ionic–covalent nature of Pb–X bonding. This characteristic orbital hybridization—where the valence band maximum is shaped by antibonding Pb-6s and halide-p interactions and the conduction band minimum by Pb-6p orbitals—is universal across compositions and sizes. It underpins bandgap tunability, defect tolerance, and strong light–matter interaction.^84,95^ A second recurring principle is the unusually strong influence of quantum confinement on excitonic behavior. Multiple hybrid DFT, GW, and BSE studies consistently report large exciton binding energies, pronounced dielectric confinement, and size-dependent optical gaps that exceed conventional particle-in-a-box models. These insights provide a coherent explanation for the narrow emission linewidths and robust excitonic signatures observed experimentally.^92,101^

A third major theme emerging from the literature is the dominant role of surface chemistry in determining both photophysical performance and stability. Across slab, cluster, and ligand-passivation DFT models, undercoordinated Pb atoms repeatedly appear as the primary origin of deep trap states, while carboxylate and phosphonate ligands show superior ability to suppress mid-gap levels and stabilize the ionic lattice. The overarching consensus is that surface energetics—more than bulk structure—govern photoluminescence quantum yield and operational lifetime in PQDs. Another fundamental insight consistently highlighted across DFT studies is the central importance of spin–orbit coupling. Inclusion of SOC is not a minor correction but a core physical requirement: it reshapes band dispersion, lowers the bandgap, modifies exciton fine structure, and can even invert bright and dark exciton ordering. These relativistic effects are intrinsic to halide perovskites, and their accurate description is necessary for interpreting optical and charge-transport phenomena.^86,89^

Beyond these areas of strong agreement, the literature also reveals several ongoing controversies and open questions. One unresolved issue concerns the origin and magnitude of Rashba splitting: while some DFT studies attribute it to intrinsic bulk inversion asymmetry, others argue it primarily arises from surface reconstruction or ligand-induced asymmetry. This uncertainty directly affects interpretations of dark-exciton behavior and thermally activated delayed fluorescence. Similarly, the precise mechanism underlying light- or field-induced degradation remains debated. Some computational works emphasize halide migration facilitated by soft phonons, whereas others identify photocharging and polaron formation as the primary drivers of instability. Although the field broadly agrees that many intrinsic defects are shallow, recent high-level DFT results show that deep traps can emerge under photocharged conditions or through specific surface reconstructions, suggesting that the notion of “defect tolerance” must be understood in a nuanced context. Finally, conflicting computational evidence exists regarding whether organic ligands simply electronically isolate the PQD core or participate in charge transfer processes that influence exciton dissociation and recombination.^94,99^

Altogether, this more integrated perspective demonstrates that the physics of perovskite quantum dots is defined not by isolated findings but by recurring conceptual patterns: the interplay between a soft lattice, strong relativistic effects, pronounced excitonic confinement, and sensitive surface energetics. By synthesizing insights from hybrid DFT, GW-BSE, nonadiabatic dynamics, and AIMD studies, a unified framework emerges that clarifies consensus views, highlights persistent uncertainties, and identifies the mechanistic foundations guiding PQD design. This concept-driven structure provides a more cohesive understanding of the field and strengthens the interpretative value of the review.

### 4.8 DFT simulation of real-world device operating conditions

Although full device-scale modeling of PQD-based LEDs, photodetectors, and sensors still requires continuum drift–diffusion or k·p methods, atomic-scale DFT coupled with dynamics has now reached the maturity to directly simulate true operating conditions—continuous illumination, applied electric bias, elevated temperature, and humidity—and quantitatively elucidate the microscopic origins of performance degradation. Under constant-current LED operation, the injected carriers generate internal electric fields of 1–5 V nm^−1^ across the few-nanometer-thick emissive layer. Recent nonadiabatic molecular dynamics studies incorporating such external fields during 100–200 ps trajectories of mixed-halide CsPb(Br_*x*_I_1−*x*_)_3_ QDs show that the bias dramatically lowers halide migration barriers from ∼0.45 eV (zero field) to below 0.08 eV, accelerating phase segregation by 4–6 orders of magnitude and faithfully reproducing the rapid red-shift and luminance decay observed in operating devices.^89,100^ The same field-driven mechanism initiates Pb^2+^ and vacancy electromigration at core–shell or ligand interfaces, nucleating voids and non-radiative recombination centers.

Under continuous illumination of 1–10 suns (relevant to photodetectors and solar cells), photo-generated hot carriers and large polarons dynamically soften the perovskite lattice. Real-time TD-DFT and surface-hopping nonadiabatic molecular dynamics at 300–350 K with multi-photon excitation capture light-soaking phenomena: transient lattice expansion, polaron-stabilized iodide clustering, and 30–70% PLQY drop over hours—in quantitative agreement with accelerated stability tests reported in the literature.^86,94^ These calculations reveal a highly mobile “soft lattice” state under illumination that is inaccessible to conventional static or ground-state DFT.

When optical pumping, bias, and junction temperature (80–120 °C) act simultaneously—as in real core–shell PQD-LEDs—Ehrenfest and nonadiabatic dynamics predict Auger-assisted ion penetration across the inorganic shell. Auger-heated electrons transiently overcome the ∼0.6 eV shell barrier, allowing gradual halide leakage that manifests experimentally as threshold current increase and EQE roll-off.^89,97^ Increasing shell thickness beyond 1.2–1.5 nm or inserting wide-gap intermediate layers raises the effective barrier above 1.0 eV, consistent with experimentally demonstrated extensions of operational lifetime beyond 10 000 h.

Finally, humidity-induced degradation under bias has been modeled by combining DFT forces with explicit-solvent *ab initio* molecular dynamics, showing water-assisted proton shuttling and ligand protonation that initiate irreversible Pb–halide bond rupture within nanoseconds.^88,102^ These examples illustrate that DFT-based frameworks—when augmented with nonadiabatic dynamics, external fields, and finite-temperature sampling—are no longer limited to equilibrium properties. They can now faithfully replicate the coupled opto-electro-thermal stress inside working devices and deliver concrete, atomic-level design rules for achieving >10 000 h stability in commercial PQD LEDs, photodetectors, and sensors.

The DFT-derived stability and interface metrics presented here enable targeted strategies that have already extended operational lifetimes from hours to >10 000 h and sensitivity limits by orders of magnitude. By combining the design rules extracted in Sections 2–4, researchers can now rationally navigate the vast compositional and structural space of PQDs with a success rate that was inconceivable five years ago.

## 5 Challenges, advantages, future directions and conclusions

### 5.1 Critical limitations of current DFT studies on PQDs and their implications

Although the present review highlights numerous impressive agreements between DFT predictions and experiment (see Table 6), it is essential to acknowledge that many of the cited results carry significant methodological uncertainties that are frequently under-discussed in the original studies. First, functional choice remains a dominant source of error. A large fraction of the literature still relies on semilocal PBE/GGA functionals for large supercells (>300 atoms), which systematically underestimate bandgaps by 0.9–1.3 eV and exciton binding energies by a factor of 3–5.^82,83^ Even when hybrid HSE06 + SOC is employed (the current gold standard), residual errors of ∼0.1–0.15 eV in bandgap and up to 30 meV in exciton energies persist, and lattice parameters are slightly overestimated, affecting calculated defect formation and migration energies.

**Table 6:** Typical uncertainties in reported DFT results for CsPbBr_3_ PQDs arising from common approximations

Property	PBE/GGA error	HSE06 + SOC residual error	Finite-size effect (<200 atoms)	Neglect of 300 K dynamics	Ref.
Bandgap	−0.9 to −1.3 eV	±0.10–0.15 eV	+0.15–0.35 eV	−0.10 to −0.20 eV	39 and 82
Exciton binding energy	×3–5 too low	±20–30 meV	±25 meV	+20–50 meV (polaron)	64 and 65
VBr formation energy	−0.4 to −0.6 eV	±0.15 eV	+0.2 eV	−0.2 to −0.4 eV (entropy)	90 and 101
Br^−^ migration barrier (NEB)	−0.2 to −0.3 eV	±0.08 eV	Minor	+0.2 to +0.4 eV at RT	89 and 100

Second, finite system size strongly influences reported values. Most studies use 2 × 2 × 2 to 4 × 4 × 4 supercells (≤160 atoms). Convergence tests show that bandgaps decrease by 0.15–0.35 eV and surface defect levels shift by up to 0.25 eV when moving to >500-atom nanoparticles—sizes that are still rarely achievable with hybrid functionals.^88,101^ Third, neglect of temperature and dynamic disorder is pervasive. Static 0 K geometry optimisations ignore soft anharmonic phonon modes and large polar fluctuations characteristic of halide perovskites. Consequently, ion-migration barriers calculated *via* nudged-elastic-band (NEB) methods (typically 0.3–0.45 eV) are widely recognised as lower bounds; explicit *ab initio* molecular dynamics at 300 K raises effective barriers by 0.2–0.4 eV and reduces defect formation energies through entropic contributions.^84,100^ Similarly, exciton-phonon coupling and dynamic Rashba effects, which can split bright/dark states by additional tens of meV at room temperature, are almost never included in the static DFT results cited throughout Sections 2–4.

Fourth, surface and ligand modelling is often oversimplified. Ideal stoichiometric terminations and frozen ligand configurations are assumed, whereas real PQDs exhibit dynamic ligand detachment, partial coverage, and solvent competition, leading to binding energy errors of 30–60% in some cases.^52,76^ These limitations do not invalidate the insights gained—hybrid DFT + SOC still offers the best available atomic-level understanding. Future progress toward truly predictive modelling will require systematic convergence with respect to functional, system size, and explicit inclusion of temperature and dynamic effects.

### 5.2 Advantages and unique strengths of DFT for PQD research

Despite these challenges, DFT remains the most versatile and insightful computational tool for PQD research, offering an unparalleled balance between physical accuracy and computational efficiency. The method's greatest strength lies in its first-principles nature—allowing direct connection between atomic structure and macroscopic properties without empirical fitting.

DFT uniquely enables quantitative visualization of charge density, bonding nature, and orbital hybridization, providing chemical intuition that is otherwise inaccessible. For perovskite nanostructures, DFT successfully reproduces experimental lattice constants, phase transitions, and electronic spectra within tight margins.^83^ It also reveals the mixed ionic–covalent character of Pb–X bonds and their sensitivity to composition, which directly dictates the optical tunability central to PQD applications. Another key advantage is the modularity of DFT frameworks: they can be extended through hybrid, *meta*-GGA, and GW-BSE approaches to progressively increase accuracy for excited states and quasiparticle energies.^94^ Such flexibility enables a continuum of methods—from routine geometry optimization to high-level excited-state analysis—within the same theoretical foundation. From an application standpoint, DFT acts as a bridge between synthesis and functionality. It predicts stable surface terminations, ligand affinities, and band alignments, guiding experimentalists toward compositions that optimize luminescence efficiency or charge transfer. In sensing and catalysis, DFT provides atomistic insight into gas adsorption, molecular recognition, and surface reaction mechanisms, allowing rational design of selective and robust PQD-based sensors.^98^

A particularly transformative strength of DFT is its compatibility with time-dependent and molecular dynamics extensions. Time-dependent DFT (TD-DFT) and ab AIMD capture photoexcitation and structural relaxation in real time, elucidating exciton migration and nonradiative decay pathways—key factors for device reliability. When coupled with nonadiabatic dynamics, DFT frameworks reveal how vibrational coherence and phonon-assisted transitions influence quantum yield, providing predictive insight into the design of efficient photonic and sensing architectures.^86,94^ The predictive power of DFT in correlating structural modifications (*e.g.*, doping, alloying, or surface capping) with performance metrics (bandgap, charge mobility, or exciton lifetime) has transformed it from a purely interpretative to a design-oriented tool. This capability to virtually “prototype” materials prior to synthesis is now central to data-driven discovery in PQD optoelectronics and sensors.

### 5.3 Five high-impact challenges that next-generation DFT/ML will solve in PQD research

The maturation of hybrid DFT, many-body perturbation theory, nonadiabatic dynamics, and especially machine-learning acceleration now places the community on the verge of solving several long-standing, high-impact bottlenecks in perovskite quantum dot (PQD) research. Below we articulate five concrete, experimentally urgent questions and explain precisely which emerging computational methodologies are now within reach to answer them definitively.

1. Can we finally predict—and completely suppress—light-induced halide segregation in mixed-halide CsPb(Br_*x*_I_1−*x*_)_3_ PQDs? Despite a decade of study, the microscopic driving force remains controversial: static DFT predicts segregation energies of only 10–30 meV per formula unit (too small to explain macroscopic phase separation), while experiments show rapid demixing under illumination. The missing physics is polaron-induced lattice softening and entropy-driven ion migration under continuous photoexcitation. Real-time nonadiabatic molecular dynamics with on-the-fly ML-accelerated HSE06 + SOC (already demonstrated on 100–200-atom supercells for >100 ps) can now capture light-induced Br/I redistribution trajectories at realistic carrier densities. These simulations will reveal whether graded halide profiles, A-site alloying (FA/Rb), or epitaxial core–shell strain can raise the effective segregation barrier above 0.8–1.0 eV, finally enabling stable red-to-green tunable LEDs and tandem solar cells.

2. What is the exact mechanism and kinetics of ligand detachment in real solvent/pH environments, and can we design ligands that survive >10 000 h under device operation? Ligand loss is the dominant degradation pathway in both LEDs and sensors, yet 99% of DFT studies still use vacuum or implicit-solvent models. Explicit-solvent *ab initio* MD is now feasible for 10–20 ns using machine-learned force fields trained on only ∼1000 DFT-MD frames (Δ-learning or M3GNet-type approaches). Such trajectories will deliver accurate free-energy barriers for protonation, desorption, and re-adsorption of oleate, phosphonate, zwitterionic, and polymeric ligands across pH 2–12 and common polar/apolar solvents. Combined with active-learning Bayesian optimization, this capability will yield the first generation of computationally pre-screened “permanent” ligands before synthesis.

3. Is fully predictive, parameter-free modelling of single-dot blinking and photoluminescence intermittency at room temperature finally achievable? Experimental on/off time distributions span nine orders of magnitude and remain unexplained by existing multiple-trap or charging models. Hybrid quantum-classical embedding methods (DFT-in-DFT, GW-in-DFT, or QM/MM with polarizable embedding) can now treat 5–8 nm QDs (∼1000 atoms) with accurate surface chemistry. When coupled with fewest-switches surface hopping and explicit Auger-assisted ionization pathways, these simulations will produce statistically converged blinking traces and power-law exponents directly comparable to single-molecule measurements. Success here would immediately guide surface passivation and core–shell strategies to push average on-fractions above 99.9%—a prerequisite for quantum light sources and ultra-stable displays.

4. Can we inversely design core–multishell heterostructures with band offsets tuned to <50 meV precision and operational lifetimes exceeding 10 000 h under heat/humidity/light stress? Today's record-breaking green LEDs (CsPbBr_3_@Cs_4_PbBr_6_/ZnS) were discovered serendipitously. High-throughput many-body GW calculations accelerated by graph neural networks trained on 10^4^–10^5^ diverse perovskite/shell interfaces can now scan thousands of combinations (Cs_4_PbBr_6_, ZnS, Cs_2_SnCl_6_, Rb_4_PbBr_6_, *etc.*) and predict type-I offsets, lattice mismatch, and interfacial defect formation energies with ±30 meV accuracy. Coupling these databases with ML-accelerated nudged-elastic-band calculations for moisture and ion penetration will deliver the first rationally designed triple-shell architectures that simultaneously maximize confinement, block degradation pathways, and maintain <1% lattice strain—a clear roadmap for commercial-grade stability.

5. Will large-scale active-learning DFT/GW finally discover lead-free, stable, bright perovskite QDs covering the entire 1.1–3.2 eV range? Over 5000 lead-free candidates have been proposed, yet none simultaneously match CsPbX_3_ in brightness, stability, and tunability. Active-learning workflows that combine uncertainty-driven DFT/GW calculations with Bayesian/global optimization have already identified several promising double-perovskite, vacancy-ordered, and 2D/0D hybrids. Scaling these pipelines to >10^6^ structures on leadership-class supercomputers (using tools such as MAST-ML, AFLOW-ML, or A-Lab-style autonomous loops) is now realistic within 2–4 years. The first experimentally validated, computationally discovered lead-free PQD with PLQY >90% and >1000 h humidity stability would immediately transform the field and eliminate toxicity concerns for consumer electronics and biomedical applications.

Solving any three of these five questions would constitute a landmark advance; solving all five would complete the transition of PQD science from trial-and-error discovery to genuine materials-by-design. The required methodological building blocks—ML-accelerated hybrid functionals, explicit-solvent long-timescale dynamics, quantum embedding, high-throughput GW, and active-learning inverse design—are no longer futuristic; they are being deployed today. The next half-decade will therefore decide whether perovskite quantum dots fulfil their promise as the universal nanomaterial platform for optoelectronics, sensing, and energy conversion.

### 5.4 Technological and practical implications

From a technological standpoint, DFT-driven insights are transforming how PQDs are engineered for real-world devices. The ability to predict and tailor band alignment, defect density, and interfacial charge transfer enables rational design of stable and high-performance PQD-based LEDs, photodetectors, and sensors. In light-emitting applications, DFT-guided defect passivation and core–shell heterostructuring have led to quantum yields approaching unity and suppressed blinking behavior. For photodetectors, simulations of carrier trapping and exciton dissociation have clarified how nanocrystal size and composition affect sensitivity and response speed. Similarly, for gas or biosensors, DFT-predicted adsorption energies and charge transfer mechanisms elucidate the selective interaction of analyte molecules with PQD surfaces.^98,100^

The broader implication is the emergence of “predictive materials design”, where DFT serves as a screening engine preceding synthesis. Instead of trial-and-error experimentation, materials can be computationally pre-validated for targeted applications—reducing cost, time, and material waste. This approach aligns with the ongoing transition toward sustainable, data-driven nanomaterials research. Moreover, as PQDs find roles in flexible electronics and wearable sensing systems, DFT insights into lattice softness, strain tolerance, and interface energetics are crucial for ensuring operational durability. The combination of DFT and experimental feedback thus enables an iterative optimization cycle: theory predicts, experiment validates, and data refines the next prediction. In this paradigm, DFT does not merely describe existing materials—it actively shapes the innovation roadmap for perovskite nanotechnology, bridging atomistic theory with functional engineering.

### 5.5 Conclusions

DFT has evolved from a theoretical framework for electronic-structure prediction into a cornerstone methodology for rational materials design. In the field of PQDs, DFT has provided unparalleled understanding of how atomic composition, quantum confinement, surface chemistry, and excitonic interactions dictate optical and electronic properties. Through its integration with hybrid, time-dependent, and molecular-dynamics extensions, DFT has elucidated the interplay between structure, dynamics, and function—bridging microscopic mechanisms with macroscopic device performance. The preceding sections of this review collectively highlight that DFT not only interprets experimental data but also predicts new materials and functionalities with remarkable accuracy. Its analytical versatility enables insights into bandgap engineering, defect passivation, doping, and stability, laying the foundation for targeted improvements in photoluminescednce, charge transport, and environmental resilience.

Looking forward, the fusion of DFT with machine learning, multiscale modeling, and emerging quantum algorithms promises to overcome current computational and conceptual limitations. This convergence will accelerate discovery of perovskite nanostructures with tailored optoelectronic characteristics for next-generation applications in light emission, energy conversion, and sensing. Ultimately, DFT stands as both microscope and compass—revealing the fundamental quantum nature of perovskite materials while guiding their evolution into technologically viable systems. By uniting first-principles rigor with data-driven adaptability, DFT continues to redefine the boundaries of computational materials science and its transformative role in modern optoelectronics.

## Conflicts of interest

The authors declare that they have no known competing financial interests or personal relationships that could have appeared to influence the work reported in this paper.

## Data Availability

No primary research results, software or code have been included and no new data were generated or analysed as part of this review.
